# Reciprocal regulation by TLR4 and TGF-β in tumor-initiating stem-like cells

**DOI:** 10.1172/JCI186923

**Published:** 2024-10-01

**Authors:** Chia-Lin Chen, Hidekazu Tsukamoto, Jian-Chang Liu, Claudine Kashiwabara, Douglas Feldman, Linda Sher, Steven Dooley, Samuel W. French, Lopa Mishra, Lydia Petrovic, Joseph H. Jeong, Keigo Machida

Original citation: *J Clin Invest*. 2013;123(7):2832–2849. https://doi.org/10.1172/JCI65859

Citation for this retraction: *J Clin Invest*. 2024;134(19):e186923. https://doi.org/10.1172/JCI186923

At the request of the corresponding author, the *JCI* is retracting this article. The authors recently became aware of incorrect images used in Figures 1A, 1C, 1E, Supplemental Figure 3D, and Supplemental Figure 7B. 

The authors apologize for these errors.

## Version 1 10/01/2024

Electronic publication

